# Pyrrole Plasma Polymer-Coated Electrospun Scaffolds for Neural Tissue Engineering

**DOI:** 10.3390/polym13223876

**Published:** 2021-11-10

**Authors:** Diana María Osorio-Londoño, José Rafael Godínez-Fernández, Ma. Cristina Acosta-García, Juan Morales-Corona, Roberto Olayo-González, Axayácatl Morales-Guadarrama

**Affiliations:** 1 Biomedical Engineering Postgraduate Program, Universidad Autónoma Metropolitana, Iztapalapa, Mexico City 09340, Mexico; dmol@xanum.uam.mx; 2 Electrical Engineering Department, Universidad Autónoma Metropolitana, Iztapalapa, Mexico City 09340, Mexico; gfjr@xanum.uam.mx; 3 Medical Imaging and Instrumentation Research National Center, Universidad Autónoma Metropolitana, Iztapalapa, Mexico City 09340, Mexico; 4 Reproduction Biology Department, Universidad Autónoma Metropolitana, Iztapalapa, Mexico City 09340, Mexico; crisbzag@gmail.com; 5 Physics Department, Universidad Autónoma Metropolitana, Iztapalapa, Mexico City 09340, Mexico; jmor@xanum.uam.mx (J.M.-C.); oagr@xanum.uam.mx (R.O.-G.)

**Keywords:** plasma polymerization, pyrrole, electrospun scaffolds, 3D cell culture

## Abstract

Promising strategies for neural tissue engineering are based on the use of three-dimensional substrates for cell anchorage and tissue development. In this work, fibrillar scaffolds composed of electrospun randomly- and aligned-oriented fibers coated with plasma synthesized pyrrole polymer, doped and undoped with iodine, were fabricated and characterized. Infrared spectroscopy, thermogravimetric analysis, and X-ray diffraction analysis revealed the functional groups and molecular integration of each scaffold, as well as the effect of plasma polymer synthesis on crystallinity. Scanning microscopy imaging demonstrated the porous fibrillar micrometric structure of the scaffolds, which afforded adhesion, infiltration, and survival for the neural cells. Orientation analysis of electron microscope images confirmed the elongation of neurite-like cell structures elicited by undoped plasma pyrrole polymer-coated aligned scaffolds, without any biochemical stimuli. The MTT colorimetric assay validated the biocompatibility of the fabricated composite materials, and further evidenced plasma pyrrole polymer-coated aligned scaffolds as permissive substrates for the support of neural cells. These results suggest plasma synthesized pyrrole polymer-coated aligned scaffolds are promising materials for tissue engineering applications.

## 1. Introduction

The nervous system, composed of the peripheral and central nervous systems, has limited self-regenerative capacity. The field of tissue engineering has made efforts to provide strategies to restore tissue structure and functionality. Biomimetic scaffolds have been designed as a strategy towards tissue regeneration, as the resultant neotissue may integrate to the host with the optimal structure for functional restoration and without rejection from the immune system. Since nerve tissue has a complex and organized structure, scaffolds comprising aligned structures for glia and neuron adhesion and migration are often used for the repair and regeneration of the nervous system [1,2].

Electrospinning is a versatile technique for the fabrication of fibrillar scaffolds [3]. Based on a relatively simple setup, nano- and micrometric fibers are prepared by electrospinning, having the capacity to support adhesion, differentiation, proliferation, and migration of cells towards nerve tissue regeneration [4]. Fibers prepared by electrospinning and its variations, such as solution blowing [5], needleless electrospinning [6,7], and in situ electrospinning [8] (among others [3,9]) have a high surface to volume ratio and endow scaffolds with pores as the fibers are deposited on the collector, which allow for cell infiltration to the scaffolds’ inner fibers [4].

The use of biodegradable and biocompatible materials is often sought for in tissue engineering, as these materials are metabolized by the host once neotissue has formed. Polylactic acid (PLA), polycaprolactone (PCL), collagen, and laminin are some examples of materials used for scaffolds in tissue engineering [4,10,11]. In particular, PLA is a biodegradable polymer which has ease of use with different fabrication techniques (like electrospinning and 3D printing) and has been used in many biomedical applications [12,13,14,15,16,17,18]. However, the interaction of PLA with biological medium is limited due to its hydrophobicity and restrictive surface for cell adhesion [15,19]. Hydrophilicity is an important property of tissue engineering scaffolds in general, as it promotes suitable interactions with culture media and tissues in vivo. Various techniques are used to enhance the hydrophilicity of scaffolds, such as plasma treatment [15,20], polymer blends [3,16] and hydrophilic material grafting [19,21], to name a few. Hence, PLA is usually complemented with other materials in order to increase tissue response.

Conducting polymers are appealing candidates in neural tissue engineering for their electrical properties, since neurons, as excitable cells, respond to electrical stimuli. Nevertheless, limitations of conductive materials include nondegradability, inappropriate mechanical properties, and their potential cytotoxicity and immunogenicity, associated with the oxidant agents used in their synthesis process [22,23,24,25]. These challenges are usually addressed by combining conductive polymers with suitable materials to form biocompatible composites while maintaining the electrical properties, such as natural polymers [22,26], hydrogels [25,27], and biodegradable polymers [11,28], among others.

Biomaterials are used as a platforms for cell support in neural tissue engineering for the treatment of traumatic and nontraumatic disease [29]. Plasma synthetized pyrrole polymer has been studied doped (pPPy/I) and undoped (pPPy) with iodine for its interactions with biological tissue, such as bone tissue [30,31], cartilage [32], pancreatic cells [33], fibroblasts [34], and neurons [35]. Moreover, pPPy/I has been thoroughly studied for its potential to protect neural tissue after damage [36,37,38,39,40,41,42]. PPPy/I has been studied as an implant in rats for the treatment of contusion and transection models of spinal cord injury (SCI), providing functional and histoarchitectural recovery for the animals after its implant. In contusion models, pPPy/I has been implanted as particles of the polymer in suspension through an injection at the injury site [36,37,38,39]. In transection models of SCI, pPPy/I has been implanted as a tablet at the transection site [40,41,42], yet other forms of implant as a treatment for traumatic and nontraumatic disease in the nervous system remain unexplored.

Plasma polymerization is a synthesis method for the fabrication of polymer films without any chemical oxidant compounds [43,44]. Plasma pyrrole polymers differ from chemically and electrochemically synthesized polypyrroles; the first consists of networked crosslinked structures, whereas the latter have a linear molecular structure. These characteristics endow plasma pyrrole polymers with appealing properties for tissue engineering applications [45]. As a result of the complex molecular structure of plasma pyrrole polymers, their electrical properties differ from chemically and electrochemically synthesized polypyrroles [43,46].

As reported by Cruz et al., the electric conductivity of the plasma pyrrole polymer (pPPy) varies between 10^−9^ to 10^−12^ S/cm. When the relative humidity (RH) of the samples increases to 90%, the conductivity increases by three orders of magnitude. A plasma pyrrole polymer doped with iodine (pPPy/I) exhibited conductivity around 10^−10^ S/cm in 30% RH and increased to 10^−6^ S/cm in 90% RH. Furthermore, in 92% RH the conductivity increased to 10^−3^ S/cm, and thus pPPy/I may be considered as a semiconductor [44].

In the present study, we designed, fabricated, and characterized electrospun scaffolds based on PLA (randomly- and aligned-oriented) coated with pPPy and pPPy/I to test their viability for neural tissue engineering applications.

## 2. Materials and Methods

### 2.1. Fabrication of Fibrous Composite Scaffolds

#### 2.1.1. Electrospinning

A solution of PLA (15% *w*/*v*) was prepared by dissolving PLA (Ingeo 3251D, Minnetonka, MN, USA) pellets in chloroform (J. T. Baker, Avantor Performance Materials, Inc., Radnor, PA, USA) and dimethylformamide (DMF, J. T. Baker, Avantor Performance Materials, Inc., PA, USA), using a ratio of 9:1. The pellets were dissolved overnight in the chloroform portion at room temperature, and the DMF was added before the electrospinning process, once the pellets were dissolved. The complete solution was homogenized using a vortex for 1 minute. The PLA solution was electrospun through a 0.6 mm internal diameter stainless steel needle and a flow rate of 2 mL/h in a TL Electrospinning and Spray Unit (TL-01 TONG LI TECH, Shenzhen, China). A high voltage power supply was used to generate an electric field of 20 kV between a collector and the needle, which were placed 22 cm apart. Aligned-fibrillar scaffolds were fabricated using a drum collector (D = 76 mm, L = 235 mm) at 2500 rpm. Random-fiber scaffolds were obtained using a plate collector. Finally, the scaffolds were dried in a vacuum oven for 4 days to remove any residual solvent.

#### 2.1.2. Pyrrole Plasma Polymer Coating

**Figure 1:**
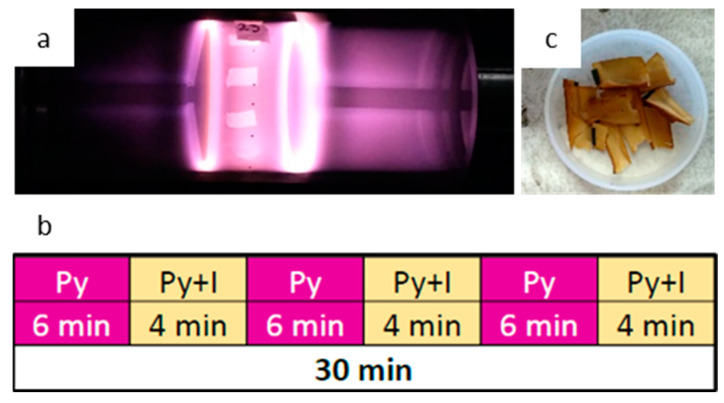
Pyrrole plasma polymer synthesis. Reactor chamber containing electrospun samples (**a**); monomer/dopant supply scheme (**b**) and resulting coated scaffolds (**c**).

PLA scaffolds were coated with plasma synthetized pyrrole polymer in a reactor as described previously [44,47]. Briefly, the scaffolds were fixed to the center of a glass cylinder, closed at both sides with stainless steel flanges coupled to electrodes fixed 10 cm apart, as shown in Figure 1. Using a vacuum pump, a pressure of 1 ± 0.1 Torr was maintained inside the reactor chamber. The glow discharges were initiated between the electrodes connected to a radiofrequency generator (Dressler Cesar 136, China) of 13.5 MHz at 30 W. Pyrrole (Py) and iodine (I) from Sigma-Aldrich vapors were supplied into the reactor chamber through connectors coupled to vessels containing the monomer and dopant. The synthesis was carried out for 30 min. For pPPy-I coating, pyrrole was kept constant while the iodine was supplied for 4 min after 6 min of pyrrole supply, as shown in Figure 1b. For pPPy coating, only Py was supplied into the chamber. Once the synthesis was completed, samples were extracted carefully from the reactor for characterization. Resulting samples are shown in Figure 1c.

### 2.2. Characterization of the Scaffolds

#### 2.2.1. Physicochemical Characterization

Physicochemical characterization was conducted by infrared spectroscopy, coupled to an attenuated total reflectance accessory (IR-ATR) in a Spectrum GX System (Perkin Elmer-DuraSamplIR II, USA), 16 scans were taken in a range of 650–4000 cm^−1^. Thermogravimetric analysis was performed in a PerkinElmer Pyris 1 TGA system, in a nitrogen atmosphere at 20.0 mL/min flowrate. A heating ramp from 30.00 °C to 600.00 °C at 10.00 °C/min was used.

Crystallinity of the scaffolds was tested by X-ray diffractometry in a BRUKER D8 ADVANCE ECO X-ray diffractometer. The degree of crystallinity was calculated using the ratio of the area under the peaks to the total area (A_T_) under the XRD pattern [48,49], which is the sum of the crystalline diffraction peak area (Ac) and the amorphous diffraction component (Aa) of the pattern (A_T_ = Ac + Aa).

Differential scanning calorimetry (DSC) tests were carried out in a TA Instruments DSC-2920 system. Between 6–8 mg of each sample were scanned at a rate of 10 °C/min, using a heating/cooling/heating program, from 30 to 230 °C. The first heating was used to erase the thermal history, and the thermal properties were obtained from the second heating.

The wettability of the fabricated scaffolds was evaluated by measuring the contact angle of a 20 µL drop of distilled water on each sample (*n* = 6).

#### 2.2.2. Morphology and Microstructure

SEM images of the as-fabricated scaffolds were obtained in a Jeol JSM-7600F scanning electron microscope. Diameter distributions of the fibers were determined taking 200 measurements per scaffold from three different frames, using ImageJ/Fiji (NIH, Bethesda, Rockville, MD, USA).

**Figure 2:**
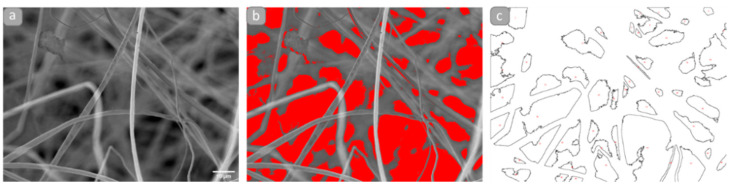
SEM images processed for bidimensional pore space segmentation. Representative images of rPLA scaffold, in (**a**) original image, (**b**) applied threshold, (**c**) detected pores.

Apparent porosity and pore dimensions were studied at the bidimensional projection from the SEM images using ImageJ/Fiji (NIH, Bethesda, Rockville, MD, USA). At least three different frames were analyzed, first by applying a contrast-based threshold where the darkest areas correspond to the pore spaces and then the particle analysis plugin. By this process, the bidimensional pore space is obtained as shown in Figure 2.

#### 2.2.3. Biological Characterization

Three dimensional cultures were conducted using the NG108-15 cell line (ATCC, HB-12317™). The NG108-15 cell line is a hybrid somatic cell type, which express properties observed in neurons and thus is used as a neuron model to study the nervous system processes such as memory, synapse, differentiation, intercellular communication, neuron membranes, trophic behavior, cellular movement, drugs, and biomaterials, among others [50,51,52,53].

The cells were cultured in standard conditions as previously reported [54]. Briefly, 10% (*v*/*v*) fetal bovine serum (FBS), 2% (*v*/*v*) HAT, and 1% (*v*/*v*) penicillin and streptomycin (100 units/mL) in DMEM were used as culture media. Cells were thawed, cultured until confluency, detached mechanically via pipetting, and counted in a hemocytometer. Cells were cultured onto the scaffolds, previously UV-sterilized, conducting a microculture method. A drop of counted cells solution was carefully deposited on each scaffold and incubated for 2 h. Culture media was then completed until the scaffolds were entirely covered. Cell cultures were maintained in a humidified atmosphere incubator at 37 °C (5% CO_2_/95% air).

For scanning microscopy imaging, 2 × 10^4^ cells were cultured on each scaffold and maintained for 15 days in standard conditions. As previously reported [54], samples were then fixed in 3.5% glutaraldehyde in 0.1 M PB at 4 °C, washed with 7.4 pH PB at 4 °C, postfixed in OsO_4_, dehydrated by immersion in ascending concentrations of ethanol in water (30–100%), and critical-point dried. Samples were mounted on SEM holders using conductive carbon tape and graphite paint, coated with gold, and visualized in a JEOL JSM-5900LV scanning electron microscope. Cell morphology and orientation of cell structures analysis was conducted in ImageJ/Fiji (NIH, Bethesda, MD, USA), with the OrientationJ plugin [55,56].

For cell viability studies, 1.65 × 10^4^ cells were cultured on each scaffold (*n* = 6) and maintained for 5 days. As reported previously [54], samples and controls (scaffolds without cells) were then transferred to a 96-well microplate, washed in PBS, and 0.5 mg/mL MTT (Sigma, St. Louis, MO, USA, M 5655) in PBS solution was added. After incubating for 2 h, DMSO (Sigma) was added and left at room temperature for 2 h in the dark. Absorbance from samples and controls was recorded in an Epoch Microplate Spectrophotometer at 580 nm and 720 nm as reference. Measurement of metabolic activity of cells on each scaffold was obtained by subtracting the absorbance at 720 nm from the corresponding absorbance at 580 nm. Since viable cells reduce MTT through metabolic activity, the absorption from dissolved MTT reduction product, which is proportional to the number of viable cells [57], was used to estimate cell viability.

#### 2.2.4. Statistical Analysis

Statistical analysis was performed in RStudio [58] and GraphPad Prism 9. Shapiro–Wilk Normality test and Kolmogorov–Smirnov test were used to explore the data. For normal data sets, ANOVA, and a Tukey HSD posthoc tests were conducted to compare the scaffolds. To compare nonnormal data, Kruskal–Wallis Rank Sum test and Dunn’s Kruskal–Wallis Multiple Comparisons posthoc test were used.

## 3. Results and Discussion

### 3.1. Physicochemical Characterization

Six types of scaffolds were fabricated and characterized: randomly oriented PLA (rPLA), aligned PLA (aPLA), pyrrole plasma polymer-coated scaffolds, random and aligned (rPLA+pPPy and aPLA+pPPy, respectively), and pyrrole plasma polymer doped with iodine-coated scaffolds (rPLA+pPPy/I and aPLA+pPPy/I).

**Figure 3:**
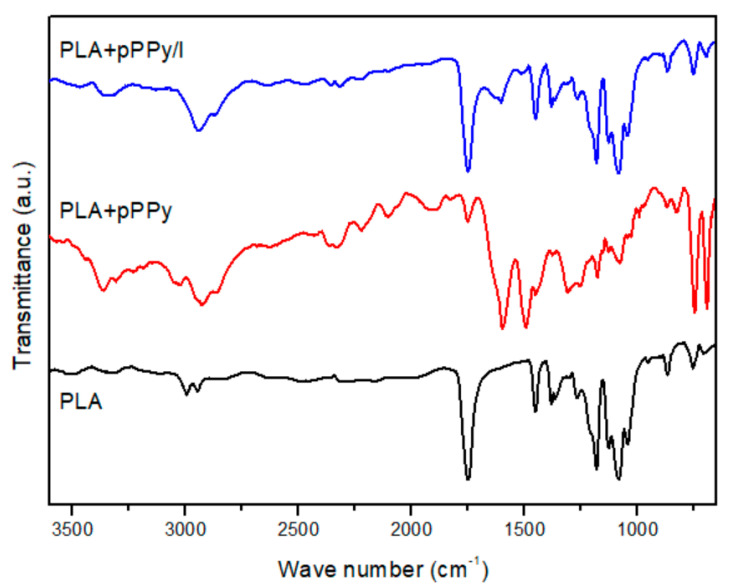
IR-ATR spectra for PLA, PLA+pPPy, and PLA+pPPy/I.

Functional groups and chemical bonds present on the molecular structure of the fibers were studied by IR-ATR. PLA, PLA+pPPy and PLA+pPPy/I spectra are shown in Figure 3. In PLA, the 3000–2500 cm^−1^, 1740–1760 cm^−1^, 1180 cm^−1^, and 1250–1050 cm^−1^ characteristic group of bands, correspond to esters (−COOH), the carbonyl group (C=O), C−O, and C−O−C respective bonds of the polymer backbone [59,60].

In contrast, PLA+pPPy spectrum shows the 3300–3400 cm^−1^ stretching vibration bands of primary and secondary amines ($-{\mathrm{NH}}_{2}$, −NH); 2960–2850 cm^−1^ bands characteristic of aliphatic carbons, 2260–2200 cm^−1^ triple bonds (C≡N and C≡C), 1660–1480 cm^−1^ −C=C− bonds and 730–675 cm^−1^ alkenes, formed from broken rings, branching, and crosslinking during the plasma synthesis. The 1250–1315 cm^−1^ band is probably due to the interaction of the material with air. PLA+PPyI spectrum shows the same characteristic bands of the pyrrole plasma polymer, with less transmittance, suggesting less pPPy/I deposit than pPPy on the PLA fibers.

**Figure 4:**
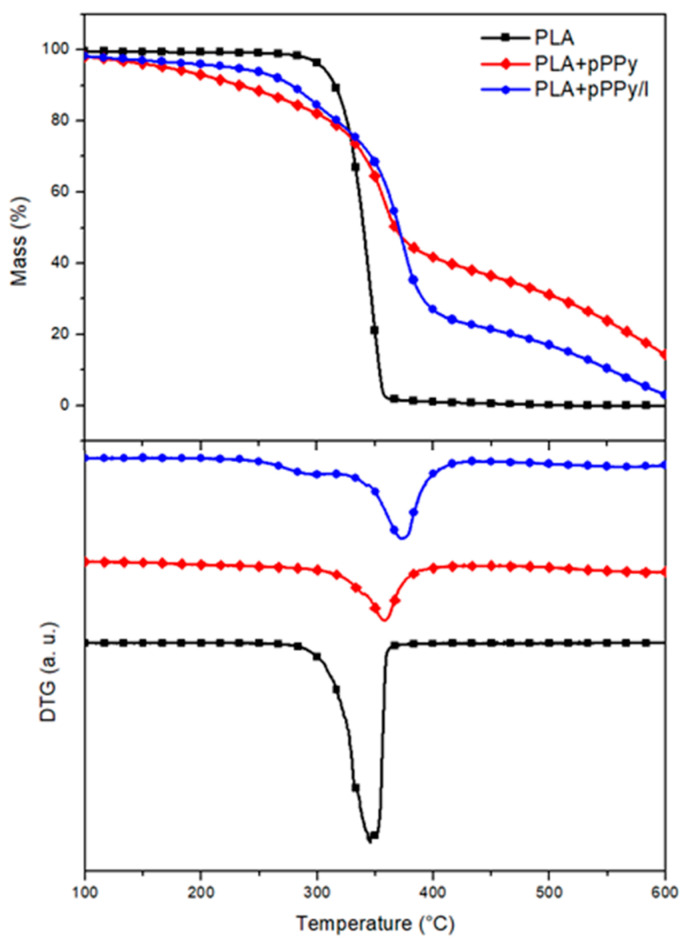
TG-DTG plots of PLA, PLA+pPPy, and PLA+pPPy/I. Thermogravimetric plots (**top**) and derivative thermogravimetry plots (DTG, **bottom**).

As shown by the TG analysis, PLA scaffold decomposition occurs in a single step, the material losses 97% of its mass between 275 °C and 370 °C (Figure 4). Coated scaffold decompositions display a 3-step process, as shown in DTG plots (Figure 4). In pPPy-coated scaffolds, a gradual mass loss begins from 155 °C and continues until the temperature reaches 290 °C. At this point, 12% of the initial mass is lost from the sample. Between 290 °C–395 °C, a 41% of mass loss takes place. Thereafter, it continues to decompose in the same gradual manner as the initial step. PPPy/I-coated scaffolds display a 17% mass loss between 217 °C and 290 °C, as clearly shown by DTG plots (Figure 4). This can be attributed to the loss of ionic iodine, not bonded covalently to the pyrrole polymer, but rather immersed in the crosslinked plasma polymerized structure [61]. Between 320 °C and 427 °C occurs the 55% mass loss of the sample. Gradual decomposition of the pyrrole polymer chains continues after 600 °C. This behavior suggests that the coating pyrrole plasma polymer starts degrading first, shifts the core PLA degradation to a higher temperature, and continues to degrade gradually thereafter, affording the fibers a higher thermal stability [34,44,62].

**Figure 5:**
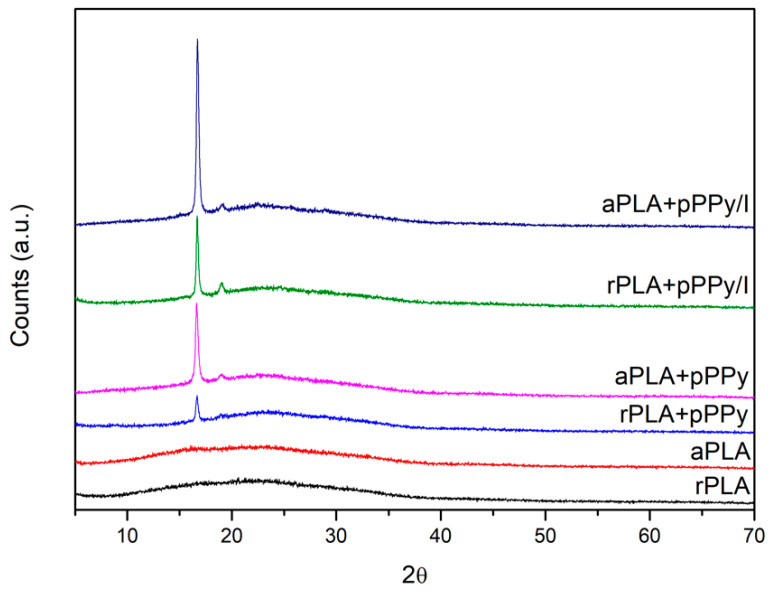
X-ray diffraction patterns of fabricated scaffolds.

XRD patterns of uncoated scaffolds display an amorphous structure in both randomly and aligned- oriented fibers (Figure 5). Coated scaffolds depict two diffraction peaks (a sharp one at 16.7° and at 19° one with less intensity), shown by the pPPy/I-coated scaffolds and aligned pPPy-coated scaffold, which correspond to (200)/(110) and (203) planes of PLA in its α-form [63,64,65]. As the process of plasma polymerization produces a crosslinked and branched structure, the patterns suggest the crystallization of PLA chains.

The degree of crystallinity was calculated from the area under the curve, using the ratio Ac/(Ac + Aa). Calculated values, as well as the intensity of the peaks (with respect to the baseline) are shown in Table 1.

Aligned scaffolds display a higher degree of crystallinity in general, as well as higher intensity of the (200)/(110) diffraction plane in coated scaffolds, suggesting that the mechanical and electrical forces during the electrospinning process contributed to a predisposition toward alignment of the PLA chains. The plasma polymerization process promoted PLA crystallization since the material was submitted to a temperature increment inside the reactor of 90 °C on average [44].

**Figure 6:**
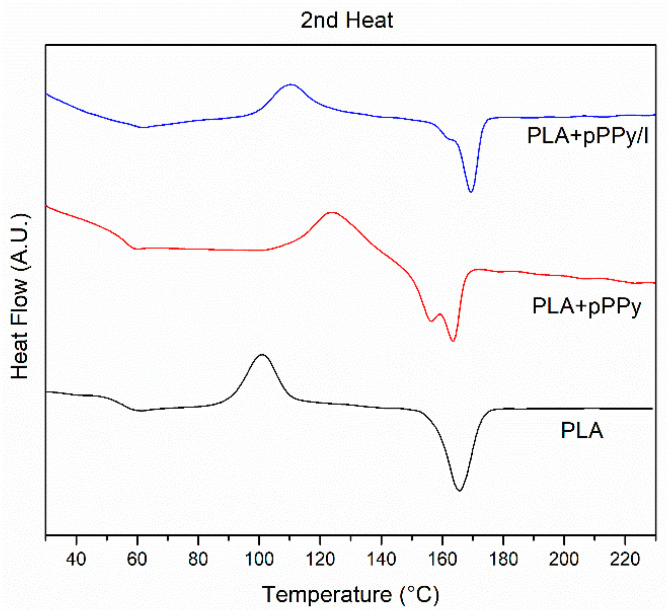
DSC curves of PLA, PLA+pPPy and PLA+pPPy/I.

DSC analysis was carried out to study the thermal properties of the fabricated scaffolds (Figure 6). The glass transition and melting temperatures are summarized in Table 2. No differences in thermal behavior were found between random and aligned fiber configurations since the DSC test reflects changes in the thermal properties of the materials. PLA displays a cold crystallization peak around 87 °C (Tcc), which is common for PLA [66]. The cold crystallization peak shifts to higher temperatures in coated scaffolds at 121.04 °C and 113.08 °C for PLA+pPPy and PLA+pPPy/I, respectively. This increase in Tcc suggests that the presence of plasma polymer hinders cold crystallization. During plasma polymerization, the PLA samples are subjected to temperatures around 90 °C inside the plasma reactor [44], which might be promoting cold crystallization of the polymer chains, and thus reflecting an increase in the degree of crystallinity and the peak displayed in XRD of coated samples [67], as shown in Figure 5.

Melting endotherms show variations in the materials. In contrast to PLA, which displays a single melting peak, PLA+pPPy shows two peaks and PLA+pPPy/I shows a shoulder at the melting peak. This behavior might be associated to a crystalline phase transition in the structure of PLA [66,68].

**Figure 7:**
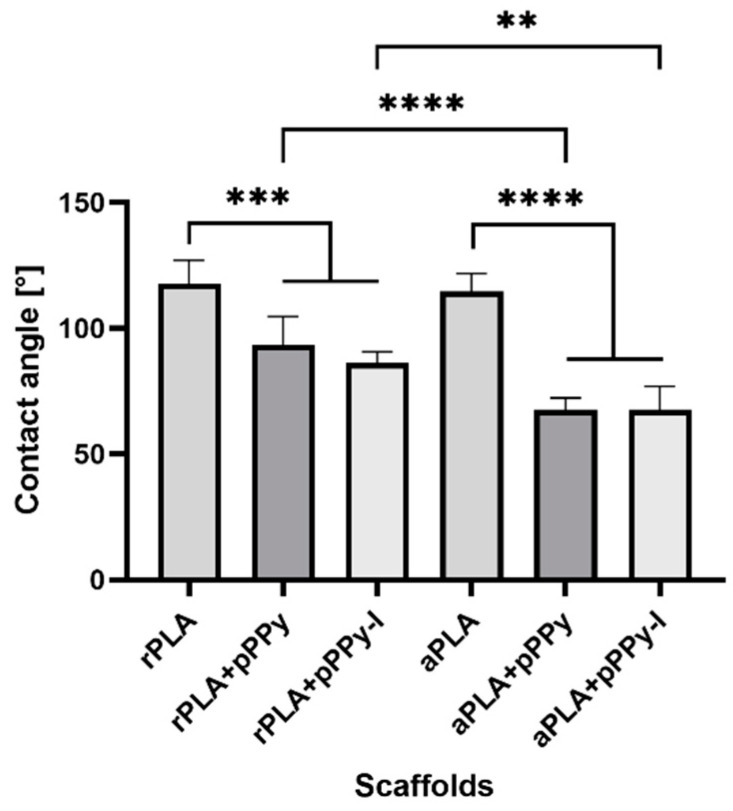
Contact angle of water on the scaffolds. Results are presented as mean ± SD, *n* = 6, ** *p* < 0.01, *** *p* < 0.001, **** *p* < 0.0001.

Figure 7 shows the contact angle measure results. As expected, PLA displays a contact angle above 100°, due to its hydrophobic nature. Coated scaffolds display lower contact angles, reflecting the fact that the plasma polymer coating promotes hydrophilicity of the scaffolds. In addition, aligned fibers contributed to lower contact angles than random fibers of coated scaffolds, suggesting that the aligned configuration facilitates the interaction between water and the inner fibers of the scaffold.

### 3.2. Morphological Characterization

**Figure 8:**
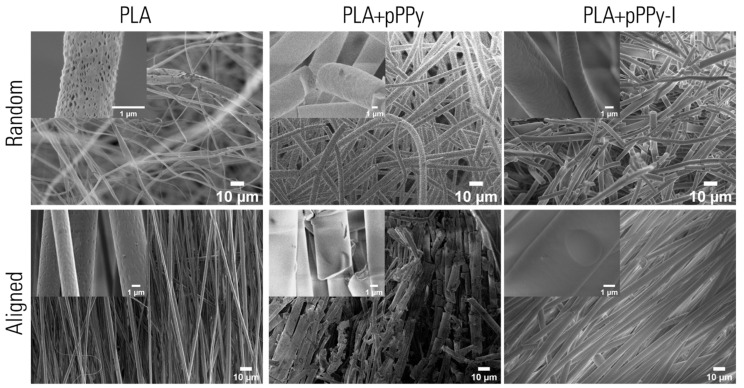
SEM images of fabricated scaffolds. Fiber microstructure shows a porous surface on PLA scaffolds whereas coated scaffolds have smooth surface fibers. Aligned scaffolds fibers display fibers oriented in a preferential direction, in contrast to randomly-oriented fibers on random scaffolds.

SEM images of the fabricated scaffolds showed the microstructure and morphology of the fibers (Figure 8). Uniform fibers free of beads or defects are observed in all samples. This is due to the polymer solution characteristics and electrospinning process conditions used, as the conductivity of the polymer solution dissipates charges resulting in the fiber elongation [69,70].

PLA-based fibers display a porous surface, whereas the coated scaffolds’ surfaces are smoothed by the pyrrole plasma polymer coating. The plasma coating was smoother in pPPy/I-coated scaffolds than in pPPy-coated scaffolds, where various fissures are present along the fibers, nevertheless, plasma polymer coating appears homogenous.

**Figure 9:**
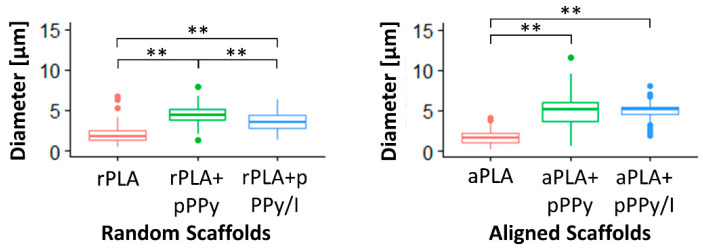
Boxplot of fiber diameter distribution. Statistical differences with the Dunn (1964) Kruskal–Wallis multiple comparison test, ** *p* < 0.01.

Statistical differences (*p* < 0.01) were found between fiber diameters, except between aPLA+pPPy and aPLA+pPPy/I scaffolds (*p* > 0.05), as shown in Figure 9. Only rPLA+pPPy displays a diameter normal distribution, whereas the other groups are log-normally distributed. The mean fiber diameters are 1.95 ± 0.950 µm in rPLA, 4.45 ± 1.08 µm in rPLA+pPPy, 3.61 ± 1.13 µm in rPLA+pPPy/I, 1.62 ± 0.741 µm in aPLA, 4.97 ± 1.64 µm in aPLA+pPPy, and 5.06 ± 0.876 µm in aPLA+pPPy/I. Average thickness of plasma polymer deposit on the PLA fibers was 2.5 µm in rPLA+pPPy, 1.66 µm in rPLA+pPPy-I, 3.35 µm in aPLA+pPPy and 3.44 µm in aPLA+pPPy-I scaffolds.

In PLA scaffolds, diameters tend to lower values with some larger diameter fibers. In coated scaffolds, as the plasma polymer formed over the fibers, adjacent fibers could become a single thick fiber. This was more frequent in aligned scaffolds and explains the widening of the diameter distribution. The presence of iodine during synthesis resulted in the destruction of smaller fibers, as shown in the distribution shift towards higher diameter values. Iodine also contributes to the formation of a more packed polymer structure [46], resulting in a smaller thickness of the polymer deposit of pPPy/I than pPPy.

In addition, statistical differences were found between random and aligned scaffolds, except between rPLA and aPLA scaffolds and rPLA+pPPy and aPLA+pPPy scaffolds (not shown), suggesting that fiber diameter is not affected by fiber orientation, but rather by plasma polymer deposition in the presence of iodine.

**Figure 10:**
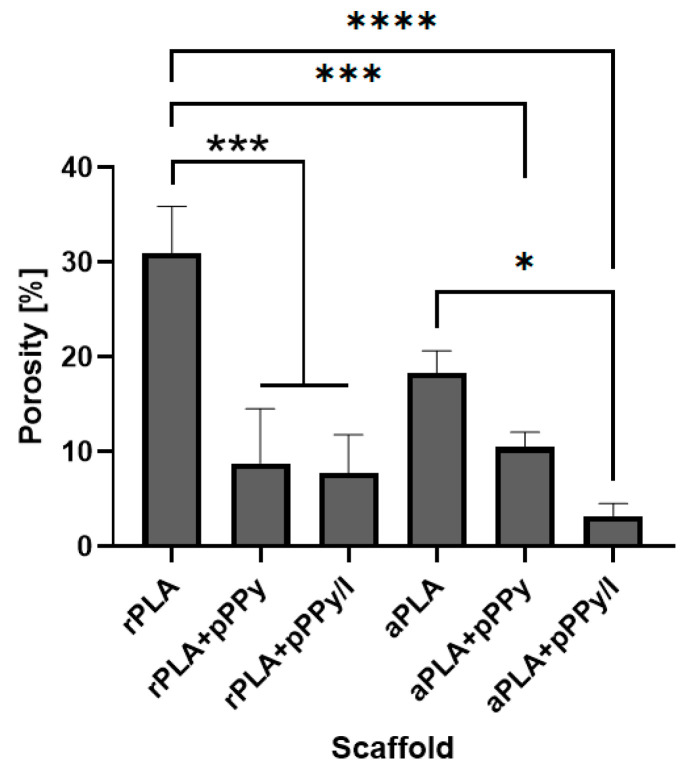
Porosity of scaffolds as-fabricated. Results presented as mean ± SD. Statistical differences with the ANOVA and Tukey multiple comparisons of means test, * *p* < 0.05, *** *p* < 0.001, **** *p* < 0.0001.

The porous microstructure of the scaffolds was maintained after the plasma polymer coating. Apparent porosity from SEM images analysis showed statistical differences between scaffolds, as shown in Figure 10. Average apparent porosity (AAP) was significantly higher in PLA scaffolds than in coated scaffolds as-fabricated. Between the PLA scaffolds no significant difference was found, suggesting that fiber orientation in this case does not influence porosity. With the plasma coating, APP is reduced due to the polymer coating over the fibers reducing the pore space in the scaffolds. No significant differences were found between coated scaffolds, suggesting once more that the plasma coating is deposited homogenously.

**Figure 11:**
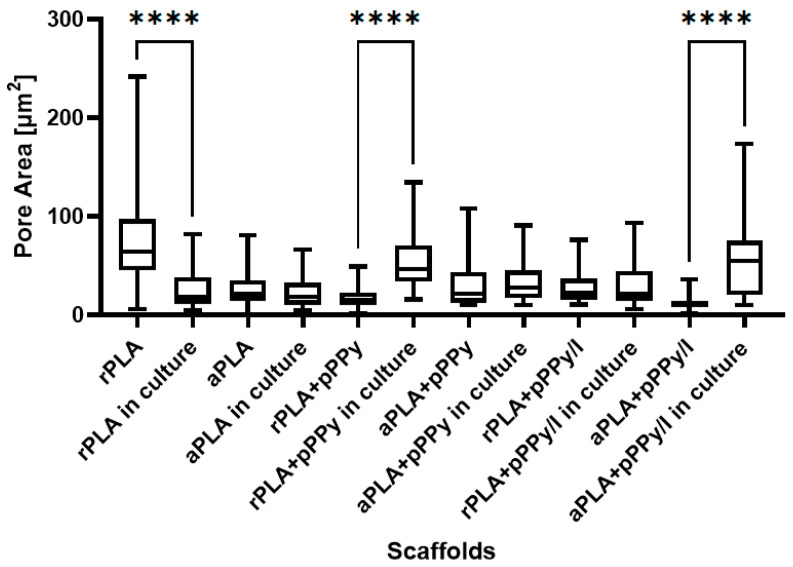
Pore area distributions of scaffolds as-fabricated and in culture, presented as box plots. Statistical differences with the Kruskal–Wallis test and Dunn multiple comparison test, **** *p* < 0.0001.

Statistical analysis of pore size showed differences between the scaffolds as-fabricated and in culture, as shown in Figure 11. Pore size significantly increased in rPLA+pPPy and aPLA+pPPy/I scaffolds in culture, suggesting that these scaffolds are expanding the porous space while interacting with cells and culture media. Since pPPy and pPPy/I surfaces are hydrophilic [45,71], scaffolds may be swelling from the media water uptake, explaining the increment in pore size in culture. However, aPLA+pPPy and rPLA+pPPy/I showed no significant differences between pore size as-fabricated and in culture, as cells proliferated mainly over the surface of the scaffolds in aPLA+pPPy scaffolds. In rPLA+pPPy/I scaffolds, although the pore size distribution shifted towards higher values in culture, the bidimensional porous space maintained the mean size in culture. However, an apparent swelling was observed on the cell culture SEM images, suggesting an increment of pore space three dimensionally.

Considering that PLA is hydrophobic [72,73], whereas pPPy and pPPy/I are hydrophilic [45,71], these results suggest that uncoated scaffolds maintained their pore size in culture and the cells occupied available pores, thus reflecting a decrement in pore size. On the other hand, an increment of pore size in coated scaffolds suggests a good interaction of the material within a biologic medium, which in culture absorbs water and swells producing an increase in porosity, allowing cells and nutrients to migrate throughout the porous structure.

### 3.3. Biological Characterization

**Figure 12:**
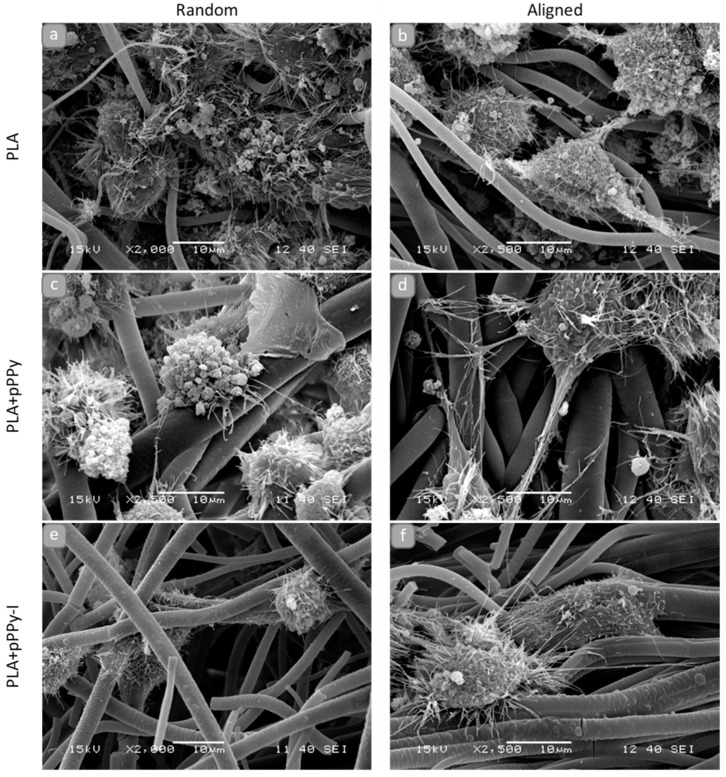
SEM images of NG108-15 cells after 15 days of culture on the scaffolds, (**a**) rPLA, (**b**) aPLA, (**c**) rPLA+pPPy, (**d**) aPLA+pPPy, (**e**) rPLA+pPPy/I, (**f**) aPLA+pPPy/I. Bar = 10 μm.

**Figure 13:**
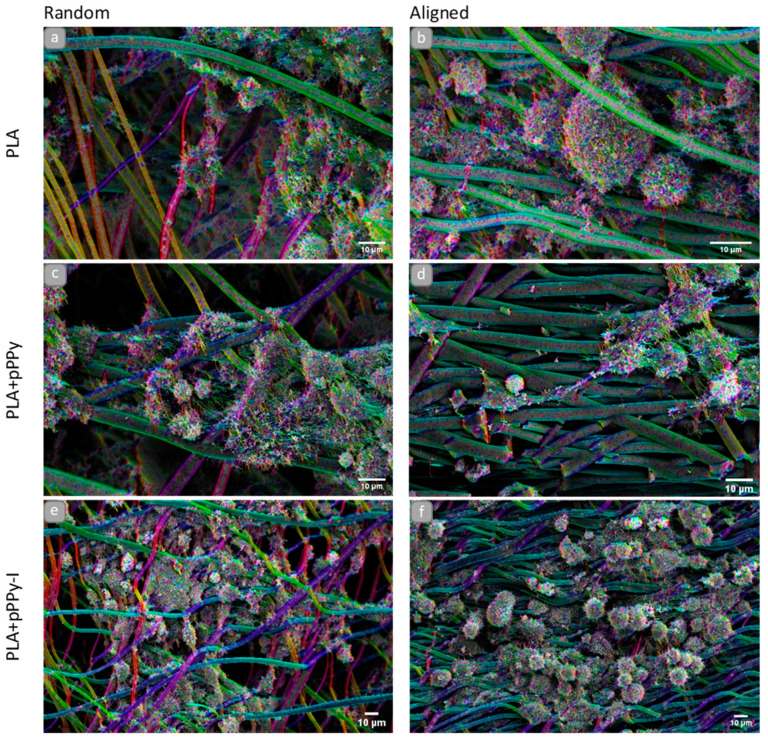
SEM images, color coded for directionality, of NG108-15 cell line on the scaffolds, bar = 10 μm. OrientationJ Fiji plugin, gaussian window σ = 0.7, min. Coherency = 2%, min. Energy = 2%. (**a**) rPLA, (**b**) aPLA, (**c**) rPLA+pPPy, (**d**) aPLA+pPPy, (**e**) rPLA+pPPy/I, (**f**) aPLA+pPPy/I.

NG108-15 cells were found anchored to al the scaffolds in general, as shown in SEM images of 3D cultures (Figure 12 and Figure 13). Randomly oriented scaffolds induced migration of the cells to inner fibers through the pores formed, as fibers were arbitrarily deposited during the electrospinning process. By contrast, aligned-oriented scaffolds afford elongated pores, which together with the organized fibers constitute a more packed 3D structure, eliciting cell growth over the surface of the scaffolds.

However, morphological differences characterized cell response to the material and the structure of the scaffolds. On rPLA, aPLA and rPLA+pPPy scaffolds (Figure 12a–c), cells with blebbing plasma membranes, rounded shapes without filopodia or retracting filopodia were found. Plasma membrane features in the form of filopodia and lamellipodia retraction and bleb formation are a distinct characteristic of cell death processes such as apoptosis and necrosis [74,75,76]. Although these processes are considered normal, they may be triggered by noxious stimuli from an unfavorable environment [74].

By contrast, cells cultured on aPLA+pPPy, rPLA+pPPy/I and aPLA+pPPy/I scaffolds (Figure 12d–f) show adequate plasma membranes, extending filopodia and forming adhesion sites on the fibers. Furthermore, neurite-like projections from cells cultured on aPLA+pPPy scaffolds, suggesting the potential of aligned pPPy-coated scaffolds for nerve cell guidance and differentiation support.

Orientation analysis on cell culture SEM images showed the orientation of cell structures over the fibers (such as filopodia projection direction and plasma membrane morphology response to the surface topography), as shown in Figure 13. Although the filopodia was projected towards adjacent cells and fibers in general, aPLA+pPPy scaffolds promoted the elongation of neurite-like structures in the direction of the fibers (Figure 13d).

Additionally, longer projections and flatter cell morphologies were identified on aPLA+pPPy scaffolds, suggesting a permissive surface for focal adhesion sites and cell attachment. Computational studies of the pyrrole plasma polymer interactions with cell membrane integrins demonstrated that the amine and hydroxy groups on the surface of the polymer have high affinity to the integrins of the cellular membrane [77], which promotes cell attachment to the coated scaffolds. Since detached cells stop proliferation processes [78], by affording cell adhesion sites, the plasma polymer coating thus promotes cell proliferation.

NG108-15 cells are rounded in shape, adherent type, extending filopodia throughout the plasma membrane surface. These filopodia have integrin adhesion receptors which bind to the extracellular matrix and move the cell [79,80]. SEM images in this work show that these cells are responding to aPLA+pPPy scaffolds, without any differentiation chemical stimulus.

Furthermore, by the implementation of the orientation analysis, slight dispersion of aligned fibers becomes evident in culture, demonstrating good mechanical response from the scaffolds to the culture conditions, such as flexibility and support for cells, while maintaining structural properties of the fibers. These features suggest pPPy- and pPPy/I-coated scaffolds have the potential to be used as implants for peripheral nerves and spinal cord regeneration, cell guidance in aligned structures, and cell therapy applications for neural tissue repair and regeneration, among others.

**Figure 14:**
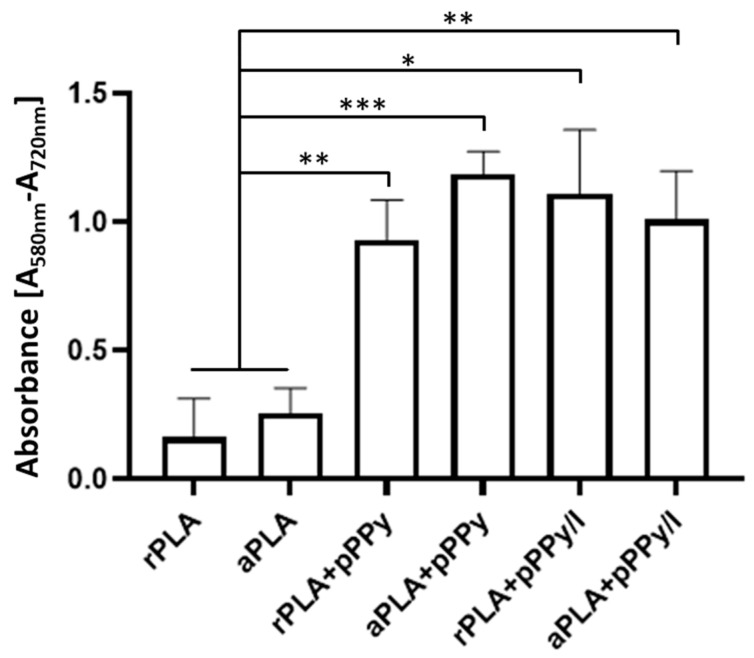
Cell viability based on the MTT assay results. Estimation of cell viability on each scaffold was based on the absorption from dissolved MTT reduction product by metabolic activity of viable cells [57]. Results are presented as mean ± SD, *n* = 6, * *p* < 0.05, ** *p* < 0.01, *** *p* < 0.001.

Cell viability was estimated by the MTT colorimetric assay, the results are reported in Figure 14. By these results, biocompatibility of the scaffolds was verified. Additionally, in accordance with SEM images, significantly higher viability was found in coated scaffolds with respect to PLA scaffolds. Overall, these results show the potential of electrospun pPPy- and pPPy/I-coated scaffolds to bind cells and promote cellular processes such as survival, migration, proliferation, differentiation, and ECM formation [81,82].

**Figure 15:**
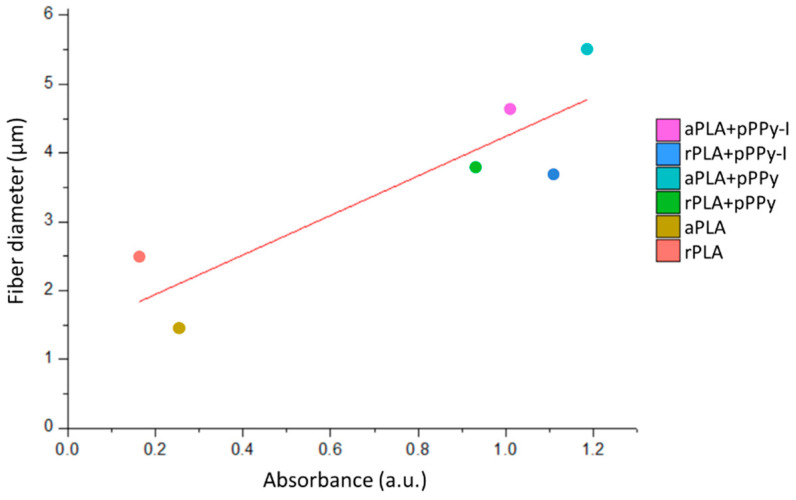
Effect of fiber diameter on cell viability. Linear correlation analysis showed a Person’s r of 0.88 between mean cell viability and mean fiber diameter.

Additionally, correlations between cell viability and the physicochemical and morphological characteristics of scaffolds were investigated. Linear correlation analysis (Figure 15) showed a Pearson’s r of 0.88 between cell viability results and fiber diameter, suggesting a dependence of adherent cells towards surface characteristics such as adhesion sites availability. Daud et al. also reported a significant difference between cell viability and fiber diameter, such that less neuronal cell viability was found in 1 µm fiber scaffolds than in 5 µm and 8 µm fiber scaffolds [83].

Evidently, fiber diameter in this case was also dependent on the composite material, as coated scaffolds afforded larger diameters together with an enriched surface composed by functional groups such as amines, aliphatic sections, nitriles, and broken rings, which promotes cell adhesion and associated physiological processes such as proliferation. Interestingly, since rPLA+pPPy and aPLA+pPPy fiber diameters were not statistically different, higher mean cell viability results in aPLA+pPPy scaffolds suggest cell preference towards aligned scaffolds.

## 4. Conclusions

Randomly- and aligned-oriented scaffolds were fabricated by electrospinning and used as core structure for novel PLA scaffolds coated with plasma synthesized pyrrole, both doped and undoped with iodine. Scaffold characterization revealed the coated scaffolds provide an enriched surface with primary and secondary amines, nitriles, and aliphatic sections, affording adhesion site availability for cell anchorage. Porosity analysis further demonstrated coated scaffolds’ ability for cell infiltration into inner fibers, due to the fibrillar porous structure and enhanced surface hydrophilicity by the plasma pyrrole polymer.

Aligned pPPy-coated scaffolds elicited neurite-like projections without the need of biochemical stimuli on the culture media, which together with the high cell viability results on these scaffolds, suggest their potential for peripheral nerve tissue engineering applications, where directionality serves as a key advantage. Both random and aligned plasma pyrrole polymer-coated scaffolds provided a permissive surface for cell survival and proliferation; nevertheless, randomly-oriented scaffolds afford high surface to volume ratio and larger pore space where higher cell infiltration and anchorage on the substrate may be key for central nervous system tissue engineering strategies. Altogether, these results suggest pPPy- and pPPy/I-coated PLA scaffolds are a promising strategy for neural tissue engineering applications.

## Figures and Tables

**Table 1:** Characteristic peak intensity and degree of crystallinity of fabricated scaffolds.

Scaffold	16.7° Peak Intensity	19° Peak Intensity	Degree of Crystallinity
rPLA	-	-	0.07
aPLA	-	-	0.14
rPLA+pPPy	629	-	0.06
aPLA+pPPy	1821	401	0.18
rPLA+pPPy/I	1669	411	0.16
aPLA+pPPy/I	3728	410	0.19

**Table 2:** Thermal properties of fabricated scaffolds.

Scaffolds	PLA	PLA+pPPy	PLA+pPPy/I
Tg (°C)	60	56.5	60
Tcc (°C)	87.44	121.04	106.73
Tm (°C)	167.58	159.47	163.25
Tm_1_ (°C)	-	153.65	158

Tg, glass transition temperature; Tcc, cold crystallization temperature; Tm, melting temperature; Tm_1_, temperature of lower melting peak.

## Data Availability

The data presented in this study are available on request from the corresponding author.
